# Effective number of breeders from sibship reconstruction: empirical evaluations using hatchery steelhead

**DOI:** 10.1111/eva.12433

**Published:** 2016-10-18

**Authors:** Michael W. Ackerman, Brian K. Hand, Ryan K. Waples, Gordon Luikart, Robin S. Waples, Craig A. Steele, Brittany A. Garner, Jesse McCane, Matthew R. Campbell

**Affiliations:** ^1^ Idaho Department of Fish and Game/Pacific States Marine Fisheries CommissionEagle Fish Genetics LabEagleIDUSA; ^2^ Flathead Lake Biological StationDivision of Biological SciencesUniversity of MontanaPolsonMTUSA; ^3^ School of Aquatic and Fishery SciencesUniversity of WashingtonSeattleWAUSA; ^4^ NOAA FisheriesNorthwest Fisheries Science CenterSeattleWAUSA; ^5^ Idaho Department of Fish and GameEagle Fish Genetics LabEagleIDUSA; ^6^ Present address: Michael W. Ackerman, Quantitative Consultants, Inc.705 S 8^th^ St.BoiseID83702USA

**Keywords:** COLONY, conservation genetics, effective population size, genetic monitoring, *Oncorhynchus mykiss*, PwoP, sibship assignment, sibship reconstruction

## Abstract

Effective population size (*N*_*e*_) is among the most important metrics in evolutionary biology. In natural populations, it is often difficult to collect adequate demographic data to calculate *N*_*e*_ directly. Consequently, genetic methods to estimate *N*_*e*_ have been developed. Two *N*_*e*_ estimators based on sibship reconstruction using multilocus genotype data have been developed in recent years: sibship assignment and parentage analysis without parents. In this study, we evaluated the accuracy of sibship reconstruction using a large empirical dataset from five hatchery steelhead populations with known pedigrees and using 95 single nucleotide polymorphism (SNP) markers. We challenged the software COLONY with 2,599,961 known relationships and demonstrated that reconstruction of full‐sib and unrelated pairs was greater than 95% and 99% accurate, respectively. However, reconstruction of half‐sib pairs was poor (<5% accurate). Despite poor half‐sib reconstruction, both estimators provided accurate estimates of the effective number of breeders (*N*_*b*_) when sample sizes were near or greater than the true *N*_*b*_ and when assuming a monogamous mating system. We further demonstrated that both methods provide roughly equivalent estimates of *N*_*b*_. Our results indicate that sibship reconstruction and current SNP panels provide promise for estimating *N*_*b*_ in steelhead populations in the region.

## 1 Introduction

Effective population size (*N*_*e*_) is among the most important parameters in evolutionary and conservation biology (Araki, Waples, Ardren, Cooper, & Blouin, 2007; Leberg, 2005; Wang, 2009). *N*_*e*_ determines the rates of genetic drift and inbreeding in a population and influences other evolutionary forces including migration and natural selection (Araki et al., 2007; Wang, 2009; Waples, 2010). *N*_*e*_ is also an important factor determining population viability (Araki et al., 2007; Frankham, Briscoe, & Ballou, 2002; Hedrick, 2005). Unfortunately, accurate estimates of *N*_*e*_ can be difficult to obtain in natural populations because many factors (e.g., abundance, sex ratio, age structure, and variance in family size) potentially reduce *N*_*e*_ to levels far smaller than census size (*N*_*C*_) estimated for the same population (Crow & Kimura, 1970; Waples, 2010). Further, it can often be difficult, time‐consuming, and expensive to collect adequate demographic data from natural populations to calculate *N*_*e*_ directly. As a consequence, many genetic methods to estimate *N*_*e*_ indirectly have been developed (as reviewed in Wang (2005)) and used widely by evolutionary and conservation biologists in recent decades (Waples, 2005).

Sibship reconstruction methods have been developed that use multilocus genotype data to identify sibling relationships among a sample of offspring and without access to parental genotypes (Emery, Wilson, Craig, Boyle, & Noble, 2001; Thomas & Hill, 2000; Wang, 2004; Wang & Santure, 2009). In other words, a sample of offspring is taken at random from a single discrete generation (ideally) of a population and any two individuals in the sample may be full‐sibs, half‐sibs, or unrelated sharing two, one, or zero parents, respectively (Wang, 2009). Sibship reconstruction attempts to identify those relationships within the sample of offspring. Importantly, accurate sibship reconstruction provides information on relatedness and variance in family size that can be used to estimate *N*_*e*_ (e.g., Perrier, Normandeau, Dionne, Richard, & Bernatchez, 2014; Richard, Dionne, Wang, & Bernatchez, 2013; Skrbinšek et al., 2012). Two *N*_*e*_ estimators using sibship reconstruction have been developed in recent years: the sibship assignment (SA) method (Wang, 2009) implemented in the program COLONY and parentage analysis without parents (PwoP; Waples & Waples, 2011).

The SA method (Wang, 2009) is based on the probability that two randomly selected individuals from a population are full‐ or half‐siblings. If *N*_*e*_ is small, the offspring have a high probability of being related. If *N*_*e*_ is large, the probability of two offspring being related is low. Wang (2009) derived an equation to calculate *N*_*e*_ as a function of the frequencies of full‐ and half‐sib dyads in a sample for a diploid population of *N*_*1*_ males and *N*_*2*_ females at each discrete generation. Denoting the probabilities of a pair of offspring being paternal half‐sibs, maternal half‐sibs, and full‐sibs as *Q*_*1*_, *Q*_*2*_, and *Q*_*3*_, respectively, he demonstrates that (eqn 10 in Wang, 2009)

$$\frac{1}{N_{e}}=\frac{1+3\alpha }{4}\left(Q_{1}+Q_{2}+2Q_{3}\right)-\frac{\alpha }{2}\left(\frac{1}{N_{1}}+\frac{1}{N_{2}}\right)$$

where α is a measurement of the deviation from Hardy–Weinberg proportions in genotype frequencies (equivalent to Wright's (1969) *F*_*IS*_ statistic). In essence, *N*_*e*_ can be estimated by the probability that a pair of offspring taken at random from a population is a half‐sib or full‐sib dyad, irrespective of their sexes and assuming there is no difference in survival between male and female offspring.

Waples and Waples (2011) presented PwoP as an alternative, but parallel, method for estimating *N*_*e*_ using information from sibship reconstruction. They developed the PwoP method when attempting to estimate *N*_*e*_ under a scenario where the parents that produced progeny are known, but no information was available about parents that produced no progeny. Standard models to calculate *N*_*e*_ require that *N*_*C*_ is known, but Waples and Waples (2011) demonstrated that no information is needed about those parents who contribute no offspring (*k*_*i*_ = 0). They show that *N*_*e*_ can simply be estimated using a vector of family sizes (*k*_*i*_) (eqn 2b in Waples and Waples (2011) which is a variant of the Crow and Denniston (1988) inbreeding effective size).

$$N_{e}=\frac{2S-1}{\frac{\sum (k_{i}^{2})}{2S}-1}$$

where *S* in the number of offspring. That is, to estimate effective population size using PwoP, all that is required is to construct the vector of parental contributions (the *k*_*i*_ values) which can be accomplished using information from sibship reconstruction analysis. See Figure 1 in Waples and Waples (2011) for a synopsis of how parental contributions (the *k*_*i*_ values) can be constructed using sibship reconstruction. Despite using genotype data, the SA and PwoP methods are in actuality hybrids of demographic and genetic approaches to estimating *N*_*e*_ in that they use sibship reconstruction to initially estimate demographic parameters (e.g., the frequencies of full‐ and half‐sib dyads or variance in family size), albeit in different forms. It is worth noting that the software COLONY implements sibship reconstruction analysis and uses the results to estimate *N*_*e*_, whereas PwoP only performs the latter and instead relies on sibship reconstruction results derived elsewhere (perhaps using COLONY).

**Figure 1:**
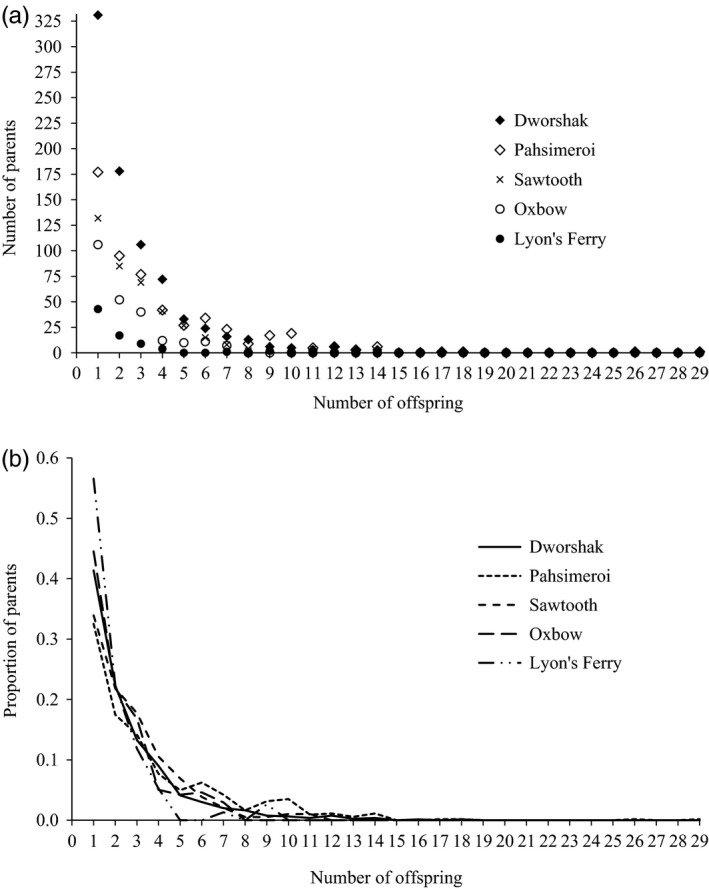
The number (panel a) and proportion (panel b) of successful parents that produced offspring and the number of offspring produced by each for five Snake River hatchery populations

Accuracy of *N*_*e*_ estimates from the SA and PwoP methods is reliant on the ability to correctly reconstruct sibling relationships. Currently, the most cited software programs that perform the generalized sibship reconstruction envisioned by the SA and PwoP methods are COLONY (Jones & Wang, 2010) and ML‐Relate (Kalinowski, Wagner, & Taper, 2006). These two programs differ mainly in the information used when predicting relatedness between pairs of individuals. COLONY potentially provides more robust (and more accurate) sibship reconstruction as it jointly considers larger patterns of relationships (e.g., between all pairs) when determining the relatedness of two individuals (Waples & Waples, 2011). In contrast, ML‐Relate determines relatedness independently for each pair of individuals, which can result in nonsensical relationship combinations (e.g., individuals A and B, and A and C are determined to be full‐sibs, but not B and C). Wang and Santure (2009) tested both programs and Waples and Waples (2011) tested ML‐Relate using simulated datasets to determine sibship reconstruction accuracy while taking into account variations in factors including sample size (relative to *N*_*C*_), the number of genetic markers (and their polymorphism), the sampling scheme used to sample offspring, and the level of relatedness within the sample of offspring. Using COLONY, Wang and Santure (2009) demonstrated that the amount of relatedness among individuals in the sample had the largest influence on the accuracy of sibship reconstruction; for instance, they observed increased accuracy with greater numbers of full‐sibs and half‐sibs present in the sample. With decreased relatedness and small family sizes, more genetic markers were needed to achieve high accuracy. Further, increased numbers of genetic markers were needed for less informative marker types. Using ML‐Relate, Waples and Waples (2011) observed a pattern of overestimation of the number of related progeny pairs, regardless of marker type (20 microsatellite loci vs. 100 SNP loci), which lead to an underestimation of the true *N*_*e*_. This bias was more pronounced as true *N*_*e*_ increased and with a small sample size (*n* = 50). This bias decreased as the number of SNPs was increased. Waples and Waples (2011) indicated that COLONY may provide more robust results than ML‐Relate, but they did not evaluate the latter's performance because of its computational intensiveness and difficulty in running large simulation studies. Recently, Wang (2016) evaluated the SA method (referred to as the sibship frequency [SF] estimator in his study) for estimating *N*_*e*_ using extensive simulations and demonstrated that the SA method is generally more robust than other single‐sample estimators under a wide range of scenarios (varied sample sizes, presence of linkage, and genotyping errors). Our current study aims to improve on these previous studies assessing the accuracy of sibship reconstruction by implementing COLONY, rather than ML‐Relate, because it more fully accounts for patterns of relatedness and by using a large empirical rather than simulated dataset.

When the SA and PwoP methods for estimating *N*_*e*_ are applied to a random sample of individuals from a single cohort in a population with overlapping generations, they provide estimates of the effective number of breeders (*N*_*b*_) of the parent generation that produced the cohort (Wang, 2016; Waples, Antao, & Luikart, 2014; Waples, Luikart, Faulkner, & Talmon, 2013). The *N*_*b*_, like *N*_*e*_, can be used to predict the genetic changes in a cohort, accounting for factors such as the variance in contribution among parents and an unequal parental sex ratio (Wang, 2009). Steelhead trout *Oncorhynchus mykiss* are a model species to evaluate the accuracy of sibship reconstruction and *N*_*b*_ estimation because large known pedigrees exist and samples can be drawn from populations of differing *N*_*b*_ sizes. In this study, we evaluated the accuracy of sibship reconstruction using a large empirical dataset of 95 single nucleotide polymorphisms (SNPs) from five Snake River (Pacific Northwest, USA) hatchery steelhead populations with known pedigrees and *N*_*b*_ (i.e., we know the true half‐sib and full‐sib relationships among the offspring). We had four objectives:

1. Evaluate the accuracy of COLONY for reconstructing full‐sibling, half‐sibling, and unrelated dyads using genotype data from *all* steelhead returning to the hatcheries in 2012 and 2013 (*n* = 4,216) that were offspring of parents spawned in 2009.
2. Compare estimates of *N*_*b*_ based on sibship reconstruction results from objective 1 to known *N*_*b*_ to examine the accuracy and precision of *N*_*b*_ estimates from the SA method when using the full offspring dataset.
3. Determine sample sizes necessary to obtain accurate and precise estimates of *N*_*b*_ by randomly subsampling offspring from each of the five hatcheries.
4. Demonstrate similarity between both the SA and PwoP methods by comparing the respective estimates of *N*_*b*_ for one hatchery (Dworshak).

For objectives 1 and 2, sibship reconstruction analyses were performed assuming both a monogamous and polygamous mating system (both sexes), and further, assuming male polygamy and female monogamy. For objective 4, sibship reconstruction analyses were limited to assuming monogamous and polygamous mating systems (both sexes). Further, for objectives 1 and 2, we examined the potential influence of varying assumed genotype error rates. Here, we provide a comprehensive assessment of the accuracy of sibship reconstruction as influenced by sample size, genotype error rates, and the choice of mating system using 95 SNPs. In total, we challenged COLONY with 2,599,961 known relationships to evaluate the accuracy of sibship reconstruction and resulting *N*_*b*_ estimates.

## 2 Materials and Methods

### 2.1 Hatchery populations

Broodstock from all Snake River hatcheries are genotyped annually as part of a parentage‐based tagging (PBT) program which, as an alternative to traditional physical tags, uses multilocus SNP genotypes to determine the origin and age of a sampled hatchery fish through parentage analysis (Steele et al., 2013). Parentage‐based tagging is typically employed to detect the presence or relative contribution of hatchery‐propagated individuals in a sample from the natural environment (Lew et al., 2015; Rechisky, Welch, Porter, Hess, & Narum, 2014). When PBT is implemented in salmonid hatcheries, the ocean‐going adults return to the hatchery annually where they are sampled, spawned, and genotyped, thereby creating large multigenerational pedigrees that provide opportunities for examining heritability of traits and reproductive success (Abadía‐Cardoso, Anderson, Pearse, & Garza, 2013) or sibship reconstruction (this study).

In this study, we used SNP data and pedigree information from five steelhead hatcheries in the Snake River basin (Dworshak, Pahsimeroi, Sawtooth, Oxbow, and Lyons Ferry) with varying abundance and *N*_*e*_. Spawning protocols at these hatcheries are intended to produce only monogamous crosses. However, shortages of returning males require these hatcheries to periodically reuse males in multiple crosses, thereby creating a small, but known, amount of polygamy in each population. Additionally, parentage results (not shown) indicate that unintentional cross‐fertilization occasionally occurs, likely during pooling of egg batches still undergoing fertilization, resulting in some 2♂×2♀ cross‐matrices. While none of the hatcheries can be expected to exclusively produce monogamous crosses, this is the mating system that predominates at these hatcheries.

Parent–offspring pedigrees were determined using parentage assignments for parents spawned in 2009 and their progeny that returned to the hatcheries in 2012 and 2013; thus, sibling relationships among the offspring are considered known. Genomic DNA extraction and SNP amplification methods are described in Steele et al. (2013). Parentage assignments were made using the program SNPPIT (Anderson, 2010) and 95 SNPs used for PBT in the Columbia River basin (Steele et al., 2013). We expect near 100% accuracy of PBT assignments (Anderson & Garza, 2006; Steele et al., 2013), and thus, consider our pedigree information to be accurate. In total, we used genetic data from 4,216 offspring returning in 2012 and 2013 produced by 2,052 unique parents spawned in 2009 (Table 1).

**Table 1:** The total number of broodstock spawned in 2009, the number of those broodstock that produced at least one offspring that returned as an adult (successful spawners), the true *N_b_* of the parent generation, the *N_b_/N_C_* ratio, the number of offspring returning to the hatchery in 2012 and 2013 from parents spawned in 2009, and the number of pairwise sibling relationships evaluated among the returning offspring for the five Snake River hatcheries included in the study

Hatchery	2009 Parents								Returning Offspring (2012 & 2013)	Pairwise Relationships
	Total Broodstock Spawned (*N*_*C*_)	Total Females Spawned	Total Males Spawned	Successful Spawners	Successful Females	Successful Males	True *N*_*b*_	*N*_*b*_ /*N*_*C*_		
Dworshak	1,873	1,096	777	799	428	371	573	.306	1,516	1,148,370
Pahsimeroi	1,279	627	652	545	273	272	353	.276	1,481	1,095,940
Sawtooth	979	489	490	389	195	194	315	.322	739	272,691
Oxbow	592	296	296	238	120	118	200	.337	400	79,800
Lyon's Ferry	215	106	109	81	41	40	58	.270	80	3,160
Total:	4,938	2,614	2,324	2,052	1,057	995			4,216	2,599,961

### 2.2 True *N*_*b*_

We first calculated the true *N*_*b*_ of the parental generation spawned in 2009 for each hatchery using the PwoP method and complete pedigree information. The *N*_*b*_ estimate from the PwoP method represents the true *N*_*b*_ when pedigree information for the population is accurate and complete (i.e., we can generate an accurate vector of *k*_*i*_ values that includes all parental contributions). As shown in Waples and Waples (2011), the resulting *N*_*b*_ values from PwoP are identical to those that would be calculated from the standard formula for inbreeding effective size (e.g., Crow & Denniston, 1988), using all parents including those that produced no offspring. Subsequent estimates of *N*_*b*_ made using the SA and PwoP methods based only on sibship reconstruction results were then compared to the true *N*_*b*_ to assess the accuracy of each method. Because PwoP does not require any information about parents that produced no surviving offspring, calculating the true *N*_*b*_ using PwoP proved easier than collecting complete demographic information from the hatchery to calculate *N*_*b*_ directly. After calculating the true *N*_*b*_ based on full pedigree information, parental genotype data were ignored for the remainder of the study, as our objective was to evaluate the ability to perform sibship reconstruction and estimate *N*_*b*_ when only offspring genotype data are available.

### 2.3 Accuracy of sibship reconstruction and *N*_*b*_ estimates using all offspring

We first assessed the accuracy of sibship reconstruction when considering genotype data from *all* offspring. Sibship reconstruction was performed using all adult steelhead returning to hatcheries in 2012 and 2013 that were offspring of adults spawned in 2009; we then compared the estimated pairwise relationships to the true relationships available from hatchery parentage pedigrees. All sibship reconstruction analyses were performed using the full‐likelihood method implemented in COLONY v2.0.5.6 (Jones & Wang, 2010) and using our set of 95 SNPs. COLONY provides an estimate of *N*_*b*_ using the SA method calculated from the frequencies of full‐ and half‐sib dyads identified in the sample. COLONY is available for download at https://www.zsl.org/science/software/colony.

For this objective, separate COLONY runs were performed for each hatchery, each containing all of the returning offspring for that hatchery. Separate runs were performed assuming three different mating systems: (i) monogamy (both sexes), (ii) polygamy (both sexes), and (iii) female monogamy but male polygamy. We evaluated the third mating system (female monogamy, male polygamy) because the monogamy option in COLONY only allows for full‐sib relationships and the polygamy option assumes a Wright–Fisher random mating model in which full‐sibs will be rare unless *N*_*e*_ is small. For most populations (including the hatchery populations in our study), the reality is likely somewhere in between. Moreover, shortages of returning males sometimes require hatcheries to reuse males. Male polygamy is also more common in natural salmonid populations as females typically deposit all of their eggs in one redd (nest), whereas males may compete and spawn with multiple females (Bentzen, Olsen, McLean, Seamons, & Quinn, 2001).

Each pair of offspring are assigned as either full‐siblings (sharing both parents), half‐siblings (sharing only one of two parents), or unrelated (sharing no parents). Again, when assuming a monogamous mating system, COLONY does not attempt to identify half‐sibling pairs, and thus, all true half‐sibling dyads are “forced” into being classified as either full‐sibs or unrelated. All COLONY runs were repeated using three different assumed genotype error rates (0.0001, 0.001, 0.01) to assess the influence of error rates on estimates of *N*_*b*_. COLONY provides an estimate of the 95% confidence limits (methods described in Wang (2009)) for each point estimate. All other default parameters in COLONY were used. In total, we performed 45 runs of COLONY (5 hatcheries × 3 assumed mating systems × 3 assumed genotype error rates) using the full offspring datasets. We compared our estimates of *N*_*b*_ from each hatchery to the true *N*_*b*_ calculated using PwoP and complete pedigree information. Throughout this study, we used the nonrandom mating *N*_*b*_ estimate from COLONY in which α (a measure of deviation from Hardy–Weinberg equilibrium) is estimated from the genotype data of the current sample (i.e., α is not assumed to be zero). We compared the sibling relationships classified by COLONY to the true relationships. In total, 2,599,961 pairwise relationships were evaluated. Of those relationships, 10,217 were true full‐sibling relationships and 4,239 were true half‐siblings; the remaining majority were unrelated pairs.

### 2.4 Sample sizes for *N*_*b*_ estimation

The aforementioned analysis is based on a best‐case scenario where data from all offspring are available to calculate *N*_*b*_. However, it is rarely logistically feasible, especially in natural populations, to sample all (or even a majority of) offspring. As is the case for most genetic *N*_*b*_ estimators, uncertainty exists around required sample sizes or the proportion of sampled offspring necessary to obtain accurate estimates of *N*_*b*_. To address this uncertainty, we iteratively and randomly sampled the offspring from each hatchery at 10%, 20%, 30%,…,90% of the total number of offspring. We performed 10 random draws at each sample size, resulting in 90 draws per hatchery. Within each draw, sampling of individuals was done without replacement; all individuals were then replaced prior to the next draw. The iterative random sampling of offspring was performed using a script written in R by the lead author (available upon request) that writes a COLONY input file after each random draw. For this objective, we performed a total of 450 runs of COLONY (5 hatcheries × 9 intervals × 10 iterations) to examine sample sizes necessary to accurately estimate *N*_*b*_. For each interval within each hatchery, we calculated bias as the percent root‐mean‐squared bias (RMSB) (Waples et al., 2014). Percent RMSB was calculated as $100\sqrt{\left[\sum {\left(\frac{{\hat{N}}_{b}-N_{b}}{N_{b}}\right)}^{2}\right]}/10$ , where ${\hat{N}}_{b}$ is the estimate of the true *N*_*b*_ and the summation is across the 10 iterations. The 450 input files were iteratively processed by COLONY using an R script (also available upon request) that executes a new COLONY run when the previous run has completed. A conservative estimate of the computation time needed for the 450 COLONY runs was 2000 hr (83 days) using a quad‐core Intel i5 3.2 GHz desktop PC with 8 GB of RAM. We mitigated this total run‐time by splitting the COLONY runs up among several computers with identical specifications. Because the study hatcheries employ a near monogamous mating system and because of difficulty in reconstructing half‐sibling relationships (results below), all COLONY runs for this objective were performed assuming a monogamous mating system. Finally, we assumed the most relaxed 0.01 genotype error rate for all analyses. *N*_*b*_ estimates from all 450 runs were then compared to the true *N*_*b*_ to evaluate the sample sizes necessary to estimate *N*_*b*_ accurately.

### 2.5 Comparison between SA and PwoP estimates of *N*_*b*_ (Dworshak)

For the last objective, we compared *N*_*b*_ estimates calculated using both the SA and PwoP methods and based on the same sets of sibship reconstruction results. Comparisons were made using both assumed monogamous (both sexes) and polygamous (both sexes) mating systems. We used our set of 90 monogamous COLONY runs for Dworshak above and also ran the same set of 90 runs except assuming polygamy. All runs were completed using the relaxed 0.01 genotype error rate. We developed a Python script that uses parentage information from sibship reconstruction for each individual (located in the .BestCluster file output from COLONY) to reconstruct the number of offspring per parent (*k*_*i*_) that is needed to create the vector of parental contributions for estimation of *N*_*b*_ using PwoP (eqn 2 in Waples & Waples, 2011). The Python script to calculate PwoP *N*_*b*_ using COLONY output files is available from the authors upon request. Finally, we compared *N*_*b*_ estimates when using the SA versus PwoP method from the 180 runs (90 runs each assuming monogamy and polygamy) of COLONY's sibship reconstruction, and assuming nonrandom mating.

## 3 Results

### 3.1 True *N*_*b*_

We calculated true *N*_*b*_ for each of the five hatchery populations using complete pedigree information and the PwoP method. The true *N*_*b*_ calculated using the PwoP method for each of the populations is as follows (from smallest to largest): Lyon's Ferry (58), Oxbow (200), Sawtooth (315), Pahsimeroi (353), and Dworshak (573) (Table 1). Figure 1 shows the family sizes (i.e., the complete vector of *k*_*i*_s) for each of the hatchery populations. The *N*_*b*_/*N*_*C*_ ratio across the five hatcheries ranged from 0.270 to 0.337 (Table 1). In the *Sample sizes for N*_*b*_ *estimation* section below, we randomly sample subsets of all offspring at 10%, 20%, 30%,…90% to estimate *N*_*b*_ using the SA method and compare those estimates to the true *N*_*b*_ to assess the sample sizes needed to accurately estimate *N*_*b*_.

### 3.2 Accuracy of sibship reconstruction and *N*_*b*_ estimates using all offspring

**Figure 2:**
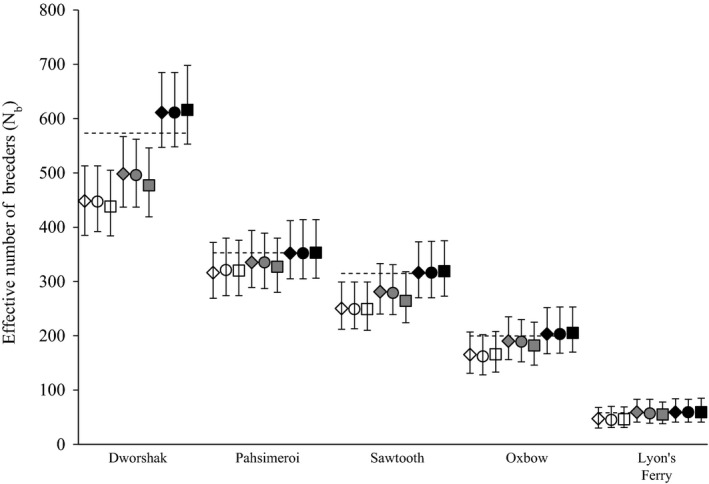
*N*_*b*_ estimates for five Snake River hatcheries assuming polygamous (open symbols), male polygamy/female monogamy (gray symbols), and monogamous (filled symbols) mating systems in COLONY. Analyses were repeated using three assumed genotyping error rates: 0.0001 (diamonds), 0.001 (circles), and 0.01 (squares). Dashed lines represent the true *N*_*b*_ for each hatchery

The program COLONY was able to identify true full‐sibling pairs with high accuracy, but did a poor job of identifying half‐sibling pairs (Table 2). Here, we summarize results only for those cases when we assumed the relaxed 0.01 genotype error rate as error rates had minimal impact on the accuracy of sibship reconstruction and *N*_*b*_ estimation (Figure 2). When we assumed a polygamous mating system, COLONY correctly identified 9,801 of the 10,217 (95.9%) true full‐sibling pairs. However, only 161 of the 4,239 (3.8%) true half‐sibling pairs were identified correctly. Of the remaining incorrectly classified half‐sibling pairs, 2,220 (52.4%) were identified as full‐siblings and 1,858 (43.8%) were identified as unrelated. As for the 2,585,505 true unrelated relationships, there were 6,204 false positives including 29 pairwise relationships falsely identified as full‐siblings and 6,175 falsely identified as half‐siblings. However, these 6,204 false positives represent merely 0.2% of the total unrelated pairs. Results across hatcheries were consistent with the exception of Lyon's Ferry (the smallest hatchery population) when assuming polygamy; the accuracy of full‐sibling reconstruction was reduced to 63 of 75 (84.0%).

**Table 2:** Pairwise relationships as estimated by COLONY v 2.0.5.6 (Jones & Wang, 2010) among steelhead returning to five Snake River hatcheries in 2012 and 2013 that are offspring of parents spawned in 2009. Results are shown when assuming monogamous (both sexes), male polygamy/female monogamy, and polygamous (both sexes) mating systems. The total column represents the true relationships (as determined by parentage); following columns represent the relationships as estimated by COLONY. Numbers in bold represent correctly estimated relationships. Results are shown using an assumed genotyping error rate of 0.01

True Relationship		Estimated Relationship							
		Monogamy		Male Polygamy, Female Monogamy			Polygamy		
Hatchery	Total	Full‐Sib	Unrelated	Full‐sib	Half‐sib	Unrelated	Full‐sib	Half‐sib	Unrelated
*Dworshak*									
Full‐sib	1,917	**1,894 (99%)**	23 (1%)	**1,858 (97%)**	45 (2%)	14 (1%)	**1,828 (95%)**	81 (4%)	8 (0%)
Half‐sib	3,124	1,780 (57%)	1,344 (43%)	1,762 (56%)	**66 (2%)**	1,296 (41%)	1,731 (55%)	**106 (3%)**	1,287 (41%)
Unrelated	1,143,329	54 (0%)	**1,143,275 (100%)**	12 (0%)	2,252 (0%)	**1,141,065 (100%)**	10 (0%)	3,158 (0%)	**1,140,161 (100%)**
Total	1,148,370	3,728	1,144,642	3,632	2,363	1,142,375	3,569	3,345	1,141,456
*Pahsimeroi*									
Full‐sib	5,895	**5,886 (100%)**	9 (0%)	**5,754 (98%)**	136 (2%)	5 (0%)	**5,695 (97%)**	199 (3%)	1 (0%)
Half‐sib	641	317 (49%)	324 (51%)	301 (47%)	**15 (2%)**	325 (51%)	288 (45%)	**29 (5%)**	324 (51%)
Unrelated	1,089,404	5 (0%)	**1,089,399 (100%)**	5 (0%)	1,152 (0%)	**1,088,247 (100%)**	5 (0%)	1,492 (0%)	**1,087,907 (100%)**
Total	1,095,940	6,208	1,089,732	6,060	1,303	1,088,577	5,988	1,720	1,088,232
*Sawtooth*									
Full‐sib	1,696	**1,695 (100%)**	1 (0%)	**1,640 (97%)**	51 (3%)	5 (0%)	**1,612 (95%)**	82 (5%)	2 (0%)
Half‐sib	73	26 (36%)	47 (64%)	25 (34%)	**4 (5%)**	44 (60%)	25 (34%)	**4 (5%)**	44 (60%)
Unrelated	270,922	15 (0%)	**270,907 (100%)**	13 (0%)	779 (0%)	**270,130 (100%)**	13 (0%)	1,045 (0%)	**269,864 (100%)**
Total	272,691	1,736	270,955	1,678	834	270,179	1650	1,131	269,910
*Oxbow*									
Full‐sib	634	**632 (100%)**	2 (0%)	**610 (96%)**	22 (3%)	2 (0%)	**603 (95%)**	30 (5%)	1 (0%)
Half‐sib	332	156 (47%)	176 (53%)	152 (46%)	**10 (3%)**	170 (51%)	146 (44%)	**16 (5%)**	170 (51%)
Unrelated	78,834	2 (0%)	**78,832 (100%)**	0 (0%)	224 (0%)	**78,610 (100%)**	0 (0%)	406 (1%)	**78,428 (99%)**
Total	79,800	790	79,010	762	256	78,782	749	452	78,599
*Lyon's Ferry*									
Full‐sib	75	**75 (100%)**	0 (0%)	**63 (84%)**	12 (16%)	0 (0%)	**63 (84%)**	12 (16%)	0 (0%)
Half‐sib	69	33 (48%)	36 (52%)	30 (43%)	**3 (4%)**	36 (52%)	30 (43%)	**6 (9%)**	33 (48%)
Unrelated	3,016	2 (0%)	**3,014 (100%)**	1 (0%)	33 (1%)	**2,982 (99%)**	1 (0%)	74 (2%)	**2,941 (98%)**
Total	3,160	110	3,050	94	48	3,018	94	92	2,974
*All Hatcheries*									
Full‐sib	10,217	**10,182 (100%)**	35 (0%)	**9,925 (97%)**	266 (3%)	26 (0%)	**9,801 (96%)**	404 (4%)	12 (0%)
Half‐sib	4,239	2,312 (55%)	1,927 (45%)	2,270 (54%)	**98 (2%)**	1,871 (44%)	2,220 (52%)	**161 (4%)**	1,858 (44%)
Unrelated	2,585,505	78 (0%)	**2,585,427 (100%)**	31 (0%)	4,440 (0%)	**2,581,034 (100%)**	29 (0%)	6,175 (0%)	**2,579,301 (100%)**
Total	2,599,961	12,572	2,587,389	12,226	4,804	2,582,931	12,050	6,740	2,581,171

When assuming male polygamy and female monogamy, full‐sibling identification improved and COLONY correctly identified 9,925 of the 10,217 (97.1%) true full‐sibling pairs. However, classification of half‐sibling pairs became poorer as only 98 of the 4,239 (2.3%) true half‐sibling pairs were identified correctly. Of the remaining incorrectly classified half‐sibling pairs, 2,270 (53.6%) were identified as full‐siblings and 1,871 (44.1%) were identified as unrelated. Regarding the true unrelated relationships, the number of false positives decreased to 4,471 (male polygamy/female monogamy) from 6,204 (polygamy both sexes). The 4,471 false positives include 31 relationships falsely identified as full‐siblings and 4,440 falsely identified as half‐siblings.

When assuming a monogamous mating system (ignoring half‐sibling relationships), full‐sibling identification improved further and COLONY correctly identified 10,182 of the 10,217 (99.7%) full‐sibling pairs. Also, when assuming monogamy, the number of false positives was greatly reduced to 78 because potential half‐sibling relationships were ignored. However, when assuming monogamy, the 4,239 true half‐sibling relationships were falsely assigned (necessarily) as unrelated or full‐siblings; of those, 2,312 (54.5%) were classified as full‐sibling pairs and 1,927 (45.5%) were classified as unrelated. Overall, assuming a monogamous mating system improved the reconstruction of full‐sibling and unrelated pairs, but with automatic and incorrect assignment of all true half‐siblings.

Point estimates of *N*_*b*_ were all within 7.4% of the true *N*_*b*_, and moreover, the true *N*_*b*_ was contained within the confidence intervals provided by COLONY for each of the five hatcheries when assuming a monogamous mating system and when all offspring were used (Figure 2). Further, estimates when assuming a monogamous mating system were better than when assuming male polygamy/female monogamy or polygamy (both sexes) despite the presence of true half‐sibling pairs in each of the offspring datasets. For four of the five hatcheries (excluding Dworshak), monogamy estimates of *N*_*b*_ from the SA method were within 2.6% of the true *N*_*b*_. For Dworshak hatchery, the *N*_*b*_ point estimate of 616 (0.01 genotype error rate) was 7.4% greater than the true *N*_*b*_ (573), but the true estimate was still contained within the confidence interval. Interestingly, Dworshak hatchery was the population for which half‐siblings were most prevalent in the data. Although the Dworshak *N*_*b*_ estimate was within 7.4% of the true *N*_*b*_, we might expect that estimates of Dworshak *N*_*b*_ would be “least” accurate given we assumed a monogamous mating system. However, the monogamous estimate was still more accurate than estimates when assuming male polygamy/female monogamy (477, 16.8% underestimate) and polygamy (438, 23.6% underestimate).

Genotype error rates had little influence on the accuracy of *N*_*b*_ estimates (Figure 2). When assuming monogamy, the largest difference among estimates occurred for Dworshak hatchery (611–616) among the three genotype error rates (0.0001, 0.001, 0.01) evaluated. The largest differences among estimates also occurred for Dworshak hatchery when assuming male polygamy/female monogamy (477–498) and polygamy (438–448); however, these difference account for <4% of the true *N*_*b*_.

### 3.3 Sample sizes for *N*_*b*_ estimation

**Figure 3:**
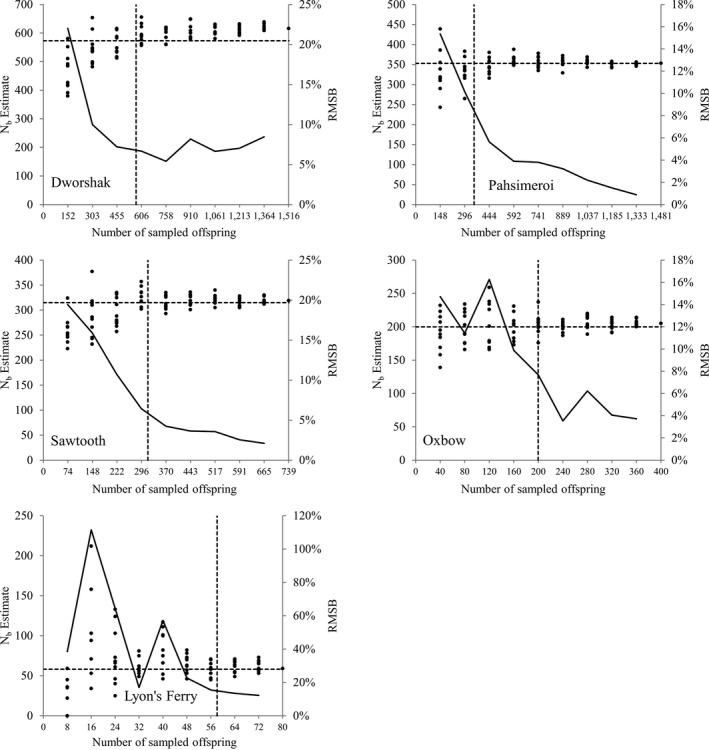
Estimates of *N*_*b*_ for five Snake River hatcheries using subsets of 10%, 20%, 30%,…100% of the total number of offspring sampled (via bootstrap resampling). Ten random draws of offspring were made at each interval for 10% to 90%. Each point represents one run of COLONY. Dashed lines represent the true *N*_*b*_. The solid line is the root‐mean‐squared bias (RMSB) among the 10 runs (secondary axis) at each sample interval

We ran 10 replicate runs using randomly drawn subsets (10%, 20%,…,90%) of offspring samples (and assuming the relaxed 0.01 genotype error rate) to evaluate sample sizes needed to accurately estimate *N*_*b*_ (Figure 3). For four of the five hatcheries (excluding Lyon's Ferry), the sample size nearest the true *N*_*b*_ resulted in a RMSB ≤ 10.1% (Dworshak = 6.7%, Pahsimeroi = 10.1%, Sawtooth = 6.4%, Oxbow = 7.7%) (Table 3). For Lyon's Ferry, the sample size nearest the true *N*_*b*_ resulted in a RMSB of 15.4%. As expected, RMSB tended to decrease as the number of sampled offspring increased and became <10% for the four largest hatchery populations and <5% for Pahsimeroi, Sawooth, and Oxbow hatcheries when sample sizes surpassed the true *N*_*b*_ (Table 3). The exception was Dworshak hatchery, where estimates became increasingly biased high when sample sizes surpassed 30% of sampled offspring (RMSB exceeded 5% at >60% sampled offspring). RMSB for Lyon's Ferry did not become <15% until ≥80% of all offspring were sampled. Table 3 shows percent RMSB as a function of percent sampled offspring for each of the hatchery populations. For the four largest hatchery populations, estimates of *N*_*b*_ were biased low when sample size was less than true *N*_*b*_ and that bias was more pronounced at lower sample sizes. When only 10% of Dworshak offspring (*n* = 151) were sampled, *N*_*b*_ estimates were biased on average 18.8% downward; however, the downward bias was reduced to 4.6% and 2.9% as the percent offspring sampled increased to 20 and 30, respectively. Oxbow, Pahsimeroi, and Sawtooth hatcheries also exhibited a downward bias at the lowest sample sizes, although not as pronounced as Dworshak; average percent bias was −4.4%, −5.5%, and −17.6%, respectively, when only 10% of offspring were sampled. However, the downward bias for the four largest hatcheries corrected as sample size approached the true *N*_*b*_. For the Lyon's Ferry hatchery with the lowest true *N*_*b*_ (58), estimates of *N*_*b*_ were generally biased high until sample size exceeded the true *N*_*b*_ and 7 of the random draws at the lowest sample size (8) resulted in estimates of infinity (data not shown in Table 3 and Figure 3). Results demonstrate that the SA method implemented in COLONY produces reasonably accurate estimates of *N*_*b*_ when sample sizes were equal to or larger than the true *N*_*b*_ and when assuming a monogamous mating system. Moreover, the precision (measured as the standard deviation among estimates) as expected improved as the number of offspring sampled increased (Table 3), although this trend was not smooth likely due to the small number of iterations (10) at each sample size (due to limitations in computation time).

**Table 3:** Summary of accuracy and precision of estimates from random sampling of offspring returning in 2012 and 2013 from parents spawned in 2009. Table shows percent and number of offspring randomly sampled; 10 iterations were performed at each interval. Summary includes the average bias of estimates relative to the true *N*_*b*_, absolute average bias, root‐mean‐squared bias (RMSB), and standard deviation (s) among estimates

Hatchery Population	Sampled Offspring (%)	Sampled Offspring	Average Bias	Average Bias (%)	Absolute Average Bias (%)	RMSB (%)	s
Dworshak	10	151	−107.9	−18.8	19.1	22.0	69.1
	20	303	−26.5	−4.6	8.9	10.0	53.6
	30	454	−16.8	−2.9	6.6	7.2	39.8
	40	606	24.1	4.2	5.1	6.7	31.3
	50	758	20.3	3.5	4.9	5.4	24.7
	60	909	38.4	6.7	6.7	8.2	28.4
	70	1061	34.8	6.1	6.1	6.7	16.4
	80	1212	38.3	6.7	6.7	7.0	13.5
	90	1364	47.6	8.3	8.3	8.5	9.8
Lyon's Ferry	10^a^	8	NA	NA	NA	NA	NA
	20	16	42.1	72.5	82.5	111.5	51.9
	30	24	15.8	27.2	49.0	64.2	35.6
	40	32	3.4	5.9	12.2	17.0	9.8
	50	40	23.5	40.4	46.7	57.2	24.7
	60	48	7.5	12.9	19.2	22.7	11.4
	70	56	−0.6	−1.0	12.9	15.4	9.4
	80	64	3.2	5.5	12.2	13.5	7.5
	90	72	3.5	6.0	8.9	12.2	6.5
Oxbow	10	40	−8.7	−4.4	12.1	14.7	29.6
	20	80	0.0	0.0	10.3	11.4	24.1
	30	120	7.7	3.9	14.7	16.3	33.3
	40	160	−2.8	−1.4	8.6	9.9	20.6
	50	200	4.5	2.3	5.4	7.7	15.4
	60	240	0.8	0.4	2.8	3.5	7.4
	70	280	7.9	4.0	5.1	6.2	10.1
	80	320	3.5	1.8	3.6	4.0	7.7
	90	360	6.0	3.0	3.0	3.7	4.7
Pahsimeroi	10	148	−19.3	−5.5	12.4	15.3	53.3
	20	296	−18.8	−5.3	8.0	10.1	32.1
	30	444	−3.9	−1.1	4.8	5.6	20.5
	40	592	7.9	2.2	2.8	3.9	11.8
	50	740	1.7	0.5	3.3	3.8	14.0
	60	888	3.1	0.9	2.5	3.2	11.6
	70	1036	4.1	1.2	1.8	2.2	6.9
	80	1184	0.1	0.0	1.2	1.5	5.6
	90	1332	−1.7	−0.5	0.7	0.9	2.8
Sawtooth	10	73	−55.5	−17.6	18.2	19.4	27.3
	20	147	−27.7	−8.8	12.9	15.9	44.1
	30	221	−20.3	−6.4	9.4	10.7	28.6
	40	295	11.3	3.6	5.0	6.4	17.7
	50	369	0.0	0.0	3.8	4.2	14.1
	60	443	4.3	1.4	3.0	3.6	11.2
	70	517	6.5	2.1	2.8	3.6	9.7
	80	591	0.4	0.1	2.3	2.6	8.5
	90	665	3.7	1.2	1.4	2.1	5.9

a Results excluded. Seven of 10 runs resulted in an estimate of infinity.

### 3.4 Comparison between SA and PwoP estimates of *N*_*b*_ (Dworshak)

**Figure 4:**
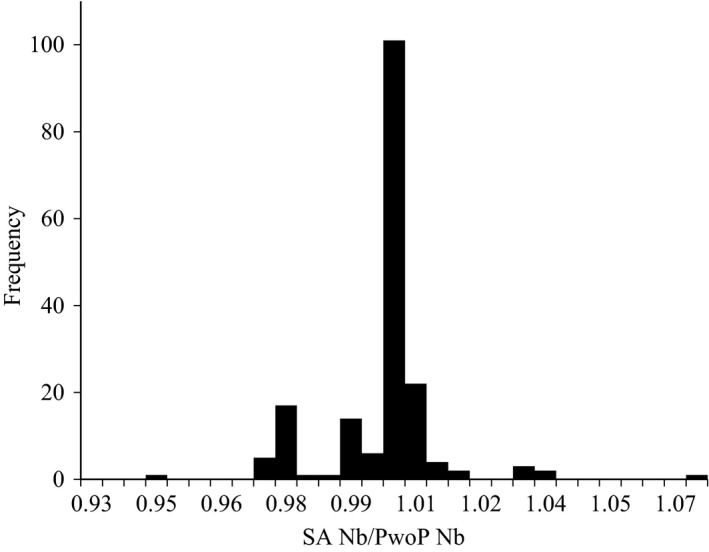
Histogram showing ratio of SA and PwoP *N*_*b*_ estimates when each are calculated using the same sets of sibship reconstruction results from COLONY. Results are shown for 180 COLONY runs of offspring from Dworshak hatchery including 90 runs assuming a monogamous mating system and 90 runs assuming polygamy

We used our previous set of 90 monogamous COLONY runs for Dworshak and also ran an additional set of the same 90 runs assuming polygamy and compared *N*_*b*_ estimates from the SA method implemented in COLONY to PwoP estimates using the same sibship reconstruction results. We found the differences in *N*_*b*_ as estimated by both methods to be slight. For 86 (47.8%) of the 180 calculations of *N*_*b*_, we found no difference in estimates (Figure 4). The greatest absolute difference between estimates was 18 which represented a 3.3% difference between estimates. Overall, estimates of *N*_*b*_ from the SA and PwoP methods never differed by >7% and differences were never >3% when sample sizes exceeded the true *N*_*b*_.

## 4 Discussion

We challenged COLONY with 2,599,961 known pairwise relationships and demonstrated that COLONY was able to accurately identify greater than 95% and 99% of true full‐sib and unrelated relationship pairs, respectively; however, reconstruction of half‐sib pairs was poor (<5% accurate). Despite poor half‐sib reconstruction, the SA method provided accurate estimates of *N*_*b*_ when sample sizes were near to or greater than the true *N*_*b*_ and when assuming a monogamous mating system which is most appropriate for the hatchery populations in this study. We further demonstrated that the SA and PwoP methods provide comparable estimates of *N*_*b*_ given the same results from sibsib reconstruction analysis. Our results indicate that sibship reconstruction and current SNP panels provide promise for estimating *N*_*b*_ in steelhead populations in the region and illustrate an approach for evaluating whether sufficient samples have been collected to accurately estimate *N*_*b*_.

We demonstrate that current full‐likelihood sibship reconstruction methods using 95 SNPs can accurately delineate full‐sib and unrelated relationships among samples of steelhead offspring and without access to parental genotypes. Of the 10,217 true full‐sib relationships among offspring within our study, 10,182 (99.7%), 9,925 (97.1%), and 9,801 (95.9%) were correctly identified when assuming monogamous, male polygamy/female monogamy, and polygamous mating systems, respectively. However, reconstruction of half‐sib relationships was poor. Among the 4,239 true half‐sib relationships, only 98 (2.3%) and 161 (4.0%) were correctly identified by COLONY when assuming male polygamy/female monogamy and polygamous mating systems, respectively. The vast majority of offspring pairs in our study were unrelated (2,585,505 pairwise relationships); >99.7% of unrelated relationships were identified correctly regardless of assumed mating system. To our knowledge, this study represents the largest evaluation of sibship reconstruction accuracy based on empirical data conducted to date. Although reconstruction of half‐sib relationships was poor, sibship‐based *N*_*b*_ estimators provided accurate estimates (<10% RMSB) in nearly every case when sample sizes were near or greater than the true *N*_*b*_ and when assuming a monogamous mating system. The relatively minor errors in estimating *N*_*b*_ despite poor half‐sib reconstruction is likely due to the fact that half‐sibs were by far the least numerous sibship class at all hatcheries (except Dworshak). In contrast, a truly random mating system would produce a large number of half‐sibs and many fewer full‐sibs. We would expect that poor resolution of half‐sib reconstruction would be a larger problem under that scenario.

Again, accuracy of half‐sib reconstruction was remarkably low in our study. The full‐likelihood sibship reconstruction method implemented in COLONY accurately identified <5% of all true half‐sib relationships in our study and was <10% accurate across all hatchery populations evaluated. In diploid species, full‐sibs each inherit alleles from two shared parents, whereas half‐sibs inherit alleles that include only one shared parent. The result is less information per genetic marker used for half‐sib reconstruction, making accurate identification of half‐sib relationships more problematic. To infer half‐sibs reliably, one needs much more marker information than that present in the 95 SNPs used in this study. Moreover, Wang and Santure (2009) demonstrated, using simulated data, that strong genetic structure (increased relatedness) among a sample of offspring resulted in much improved sibship reconstruction. In our study, the frequency of true sibling pairs was low, whereas unrelated pairs were frequent (i.e., weak genetic structure), and so it is not surprising that half‐sib reconstruction was poor given the low degree of relatedness among the hatchery offspring analyzed and the relatively few SNPs (95) used for sibship reconstruction. Further research is needed to evaluate how the number and diversity of SNPs and the degree of relatedness among a sample of offspring jointly influences the accuracy of half‐sib reconstruction. Moreover, in a scenario with relatively few half‐sib pairs (as in our study), perhaps methods that jointly consider all pairwise relationships simultaneously (e.g., COLONY) have a difficult time identifying half‐sib pairs and methods that strictly evaluate pairwise relationships (e.g., ML‐Relate) may prove beneficial; more research is needed in this area. Although out of the scope of the current study, jointly analyzing sibship and parentage by including genotype data for candidate parents (when available) would almost certainly ameliorate difficulties in half‐sib reconstruction.

The SA and PwoP *N*_*e*_ estimation methods each have several appealing traits. First, both can be applied to a single sample of offspring in the absence of parental genotypes. This is important especially for natural populations where it can be logistically difficult to obtain samples from both parents and their offspring or where it is difficult to obtain samples separated by several generations. Second, both methods avoid common assumptions that can limit application of other *N*_*e*_ estimation methods. One common assumption often required for *N*_*e*_ estimation is that of random mating, which is particularly important to heterozygote methods and (to a lesser degree) linkage disequilibrium methods. Violation of the random mating assumption can result in levels of heterozygote excess or linkage disequilibrium that is different from random mating expectations and lead to biased estimates of *N*_*e*_ using these methods. The SA and PwoP methods are less susceptible to violations of random mating (Wang, 2009; Waples & Waples, 2011). Another common assumption is that of an isolated population closed to immigration; violation of this assumption can lead to biased estimates of *N*_*e*_ from the temporal method (Wang & Whitlock, 2003) or the linkage disequilibrium method (England, Luikart, & Waples, 2010; Waples & England, 2011). The SA and PwoP methods do not require expectations of a closed population with no immigration. Rather, when individuals from more than one population appear in the sample of offspring, any immigrants would presumably be determined to be unrelated to the local sample, which would tend to increase *N*_*e*_. This would reflect the reality that the sample of offspring is produced by more parents than are present in the local population (Waples & Waples, 2011). However, if one is interested in estimating only the local *N*_*e*_, the presence of immigrants may produce an undesired upward bias in *N*_*e*_ estimates. Third, the sibship reconstruction required to estimate *N*_*e*_ via the SA and PwoP methods provides additional demographic information (e.g., distribution of family sizes) as a “byproduct” that is not provided by other methods. Finally, Wang (2009, 2016) demonstrated that the SA method is more accurate than some other genetic methods (heterozygote excess and temporal) at estimating *N*_*e*_ in most circumstances.

Perhaps, the primary disadvantage of the SA and PwoP methods is the computation time required by COLONY which employs the currently best maximum‐likelihood sibship reconstruction method available. In total, our study required greater than 3 months of computation time. We were able to partially mitigate for this total run‐time by splitting COLONY runs among several computers with the same specifications. However, many researchers may not have the opportunity to employ several computers or computationally efficient servers. Moreover, the COLONY input files required for batch processing contain complex formatting and several parameters than can make it difficult to create several (dozens or hundreds) of input files at a time. Waples and Waples (2011) used ML‐Relate for evaluations of the PwoP method to reduce computation time; however, ML‐Relate independently estimates the relationship between each pair of offspring rather than jointly considering larger patterns of relationship. Thus, ML‐Relate is theoretically less accurate than COLONY for sibship reconstruction (although we did not perform a comparison in our study) and ML‐Relate can create nonsensical pedigrees because it does not jointly consider relationships. Anderson and Ng (2016) explored Bayesian pedigree inference via a factor‐graph representation as an alternative to the maximum‐likelihood methods employed by ML‐Relate and COLONY. They demonstrate their method to be computationally feasible, while also providing more accurate pedigree reconstruction and better estimates of uncertainty for assigned pairwise relationships than COLONY. Anderson and Ng (2016) only evaluated full‐sibling relationships in their study; however, they are further investigating and developing their methods to incorporate half‐sibling reconstruction. Further development of Bayesian pedigree inference via factor‐graph representation provides promise for more computationally efficient and accurate sibship reconstruction which would benefit both the SA and PwoP *N*_*e*_ estimators.

We evaluated the influence of varying genotype error rates on the accuracy of sibship reconstruction and the resulting impact on *N*_*b*_ estimates. According to the COLONY user guide (available with software download), the error rate incorporates the allelic dropout rate plus other kinds of genotyping errors (including mutations) of the marker. We evaluated three error rates (0.0001, 0.001, and 0.01). These error rates translate to one error per 10,000 genotypes to one error per 100 genotypes. As noted in the results, varied genotype error rates had little influence on the accuracy of *N*_*b*_ estimates despite a 100‐fold change in assumed error rates. Within each hatchery and mating system scenario, *N*_*b*_ estimates never varied by greater than 7% (55 [0.01] vs. 59 [0.0001], Lyon's Ferry hatchery, male polygamy/female monogamy). The largest absolute difference in estimates occurred within Dworshak hatchery when assuming male polygamy/female monogamy. The *N*_*b*_ estimate was 498 when assuming a 0.0001 genotype error rate versus 477 assuming a 0.01 error rate; however, this difference only represents 3.7% of the true *N*_*b*_ for Dworshak hatchery. The accuracy of full‐sib reconstruction ranged from 95.0% (0.0001 error rate) to 95.9% (0.001 and 0.01 error rates) when assuming polygamy. Half‐sib reconstruction remained <3.8% accurate regardless of genotype error rate. Note that the computation time for the full‐likelihood sibship reconstruction method (the default method in COLONY) suffers from increased genotype error rates; the higher the error rate, the slower the computation time. If computation time is a concern, then one might choose to assume a low genotyping error rate and expect limited influence on sibship reconstruction and *N*_*b*_ accuracy. However, if computation time is not a concern, then we suggest assuming a more relaxed genotype error rate.

We demonstrated that the two sibship‐based *N*_*e*_ estimators we evaluated were accurate for the five Snake River hatchery steelhead populations given sufficient samples and when assuming a monogamous mating system. Each of these hatcheries employs predominately monogamous crosses (but see Section 2.1 for a more detailed explanation of mating systems employed at study hatcheries), and thus, assuming a monogamous mating system was most appropriate for our study. However, our desire is to ultimately apply sibship‐based (or other single‐sample *N*_*e*_ estimators) to natural populations where mating systems are typically less understood. Data are sparse regarding the extent of polygamy that occurs in natural populations of *O. mykiss* containing predominately individuals with an anadromous life‐history. Across their range, anadromous steelhead are often iteroparous (may undergo multiple migrations to the sea to optimize growth and multiple upriver migrations to spawn); the goal of anadromy and iteroparity is to increase lifetime reproductive success. However, in the Snake River basin, steelhead iteroparity rates are very low (0.5% to 1.2%, Keefer, Wertheimer, Evans, Boggs, & Peery, 2008; Matala et al., 2016) primarily due to long migration distances and difficulties with navigating the Columbia River and Snake River hydropower systems. Thus, Snake River steelhead are considered primarily semelparous (undergo only one seaward and one upriver spawning migration); the vast majority of Snake River steelhead only experience one spawning event in their lifetime. Moreover, female steelhead typically deposit all of their eggs in one redd (nest). Males have the opportunity to spawn with multiple females, however typically spawn with only one female; subsequent spawning events with any additional females generally result in lower reproductive success.

The largely semelparous and anadromous life histories of Snake River steelhead promote a primarily monogamous mating system in the natural environment. However, anadromous Snake River steelhead populations often occur in sympatry with resident individuals (rainbow trout). Resident *O. mykiss*, unlike steelhead, do not undergo a seaward migration to optimize growth. Rather, resident individuals are generally males that attempt to increase lifetime reproductive success by avoiding potential mortality associated with migration to the ocean and back. Resident males may spawn across multiple years and “sneak” onto a female steelhead's redd to spawn. The presence of resident individuals in a population may increase polygamy in an *O. mykiss* population, but residents occur in varying (and often unknown) proportions in *O. mykiss* populations. Further research is needed to better understand mating systems in natural *O. mykiss* populations containing both anadromous and resident individuals across the species’ range and how varied mating systems may impact sibship‐based *N*_*e*_ estimators. Further simulation studies may elucidate how mating systems may impact single‐sample *N*_*e*_ estimators.

Assuming a monogamous mating system resulted in the most accurate estimates of *N*_*b*_ for each of the hatchery populations, whereas assuming polygamy (for males or both sexes) generally resulted in an underestimate of the true *N*_*b*_ (Figure 2). When sample size *n* is small relative to the true *N*_*b*_ (i.e., the ratio of *n*/*N*_*b*_ is low), as was the case in our study, Wang (2016) noted that true sibling frequencies (full‐sibs and half‐sibs combined) are expected to be low relative to the frequencies of nonsibs. Under this scenario, one might expect many more type I errors (unrelated pairs assigned as false sibs) than type II errors (related pairs assigned as false nonsibs), simply due to the ratio of unrelated to related pairs present in the sample (Wang, 2016). In our study, the ratio of true unrelated to related pairs was >175:1. Using the definitions of type I and type II errors defined by Wang (2016), the frequency of type I versus type II errors observed in our study for each of the three mating systems evaluated and using the full offspring data sets were as follows: monogamy (78 type I; 1,962 type II), male polygamy/female monogamy (4,471 type I; 1,897 type II), polygamy (6,204 type I; 1,870 type II). In other words, the number of type I errors greatly outnumbered the number of type II errors when assuming male polygamy/female monogamy or polygamy (both sexes), whereas type II errors outnumbered type I errors when assuming monogamy. The overestimate of the number of related individuals present in the sample when assuming polygamy likely explains resulting underestimates of *N*_*b*_.

In nearly every case, a sample size near or greater than the true *N*_*b*_ resulted in a RMSB of <10% for estimates of *N*_*b*_ in each of our hatchery steelhead populations. Moreover, sample sizes below the true *N*_*b*_ generally resulted in a downward bias in *N*_*b*_ estimates. For the three largest hatchery populations (Dworshak, Pahsimeroi, Sawtooth), a sample size approximately half of the true *N*_*b*_ resulted in a downward bias of near 5%–10%. The requirement of a sample size near the true *N*_*b*_ and a downward bias in estimates at low sample sizes is similar to findings by England, Cornuet, Berthier, Tallmon, and Luikart (2006); however, see Waples (2006) for a bias correction for estimates of *N*_*e*_ from linkage disequilibrium methods. The exception to this rule was the Lyon's Ferry hatchery, the smallest hatchery population in the study with a true *N*_*b*_ of 58. At the lowest sample sizes (16–40), there was an upward bias in *N*_*b*_ estimates; however, *N*_*b*_ estimates still approximated the true *N*_*b*_ when sample sizes approached and exceeded the true *N*_*b*_. We acknowledge that researchers often only have 30–100 samples from a population for estimating *N*_*b*_. Aside from Lyon's Ferry (where all sample sizes were <100), in only three cases did we have a sample size of less than 100; when 10% and 20% of Oxbow hatchery offspring were sampled and when 10% of Sawtooth hatchery offspring were sampled. These sample sizes resulted in an average percent bias of −4.4%, 0.0%, and −17.6%, respectively. Although small sample sizes resulted in little to no bias, we emphasize that low sample sizes can cause (in some cases severe) downward bias when sample sizes are low relative to the true *N*_*b*_ of the population. Waples and Waples (2011) demonstrated that the PwoP method is unbiased regardless of sample size. However, that accuracy of estimates from PwoP depends on the accurate reconstruction of the pedigree. Therefore, the observed pattern of downward bias in *N*_*b*_ estimates at low sample sizes in our study is likely due to the effects of sample size on sibship reconstruction in COLONY.

For natural populations where the true *N*_*b*_ is unknown and where an initial estimate is desired (say, in the first year of a study), we recommend that researchers attempt to achieve sample sizes beyond a best estimate of the true *N*_*b*_, perhaps a few to several hundred samples depending on population size. By doing so, this would allow one to iteratively subsample individuals (as done in this study) and plot estimates at each sample size to examine whether estimates achieve an asymptote at increasing sample sizes. If estimates do not achieve an asymptote, this may suggest too few samples were collected. Alternatively, if an asymptote in estimates is achieved, then *N*_*b*_ estimates at the largest sample size should be accurate (assuming sibship reconstruction is reasonably accurate and the mating system is well understood). We are currently developing software that will iteratively subsample individuals, provide an estimate of *N*_*b*_ for each subsample, plot estimates of *N*_*b*_ at varying sample sizes, and provide guidance on whether adequate samples were taken to achieve an accurate estimate of *N*_*b*_.

The SA and PwoP methods produced essentially equivalent estimates of *N*_*b*_ when provided the same sibship reconstruction results from COLONY. This is because the two estimators are conceptually the same, but in different forms. The SA method estimates *N*_*e*_ using sibship frequencies, while PwoP uses variance in family size. However, sibship frequency and family size variance are essentially interchangeable, as shown in the derivation of the SA estimator by Wang (2009). In his derivation, Wang (2009) first expressed the SA equation in terms of family size variance before transforming the equation to be in terms of sibship frequencies. The only real difference between the two estimators is regarding the assumption of random mating. PwoP assumes random mating, whereas SA uses the deviation from Hardy–Weinberg equilibrium (expressed as α) to account for nonrandom mating. Slight differences in estimates between the two estimators are largely due to nonrandom mating. In the Supplementary Material provided, we further demonstrate similarities between the two estimators. Because of the similarities, we consider our study to be a rigorous evaluation of both sibship‐based estimators.

The SA and PwoP estimators are subject to the same biases, and the accuracy of *N*_*b*_ estimates from each is equally reliant on the accuracy of the sibship reconstruction results used. In this study, we used a panel of 95 SNPs that were developed for PBT of hatchery populations in the Snake and Columbia River basins. However, our laboratory and others involved in salmonid research have recently implemented high‐throughput genotyping methods that provide the ability to screen many individuals at a couple‐ to several‐hundred or even thousands of SNPs at reduced genotyping cost. These methods include Genotyping‐in‐Thousands by Sequencing (GT‐seq, Campbell, Harmon, & Narum, 2015) and RAD Capture (Rapture, Ali et al., 2016). With the potential for high‐throughput genotyping at reduced cost, further research is needed to determine the number and diversity of SNPs that would optimize sibship reconstruction including reconstruction of half‐sib relationships. We are currently at a crossroads with the opportunity to develop SNP panels that can achieve multiple objectives (e.g., single‐parent parentage, genetic stock identification, hybrid detection, population genetic structure) including sibship reconstruction. Moreover, further development of Bayesian methods of pedigree inference (Anderson & Ng, 2016) may improve the accuracy of sibship reconstruction and make batch processing of sibship reconstruction analyses more computationally feasible.

Both sibship‐based estimators of *N*_*b*_ (SA and PwoP) reliably estimated the true *N*_*b*_ in all five hatchery populations when sample sizes were equal or larger than the true *N*_*b*_ and when assuming monogamy. However, if *N*_*b*_ estimates are larger than the sample size used for sibship reconstruction, researchers might expect that *N*_*b*_ estimates are downwardly biased. To evaluate for potential bias, we propose a framework, whereas researchers could potentially iteratively subsample individuals to assess whether small sample sizes are resulting in biased estimates of *N*_*b*_ (e.g., England et al., 2010). Our study suggests the SA and PwoP methods provide reliable estimates of *N*_*b*_ in populations as long as the mating system is fairly well understood, for example, >80%–90% monogamy actually exists when we assume monogamy, and when using 95 SNP loci. Lessons learned in this study may be applied to future research on wild populations: 1) A sample size near or above the true *N*_*b*_ is necessary to provide an accurate estimate using sibship reconstruction to estimate *N*_*b*_, and 2) we can iteratively subsample offspring and assess whether we have achieved an asymptote in our estimates to help determine whether we have obtained an adequate sample size (England et al., 2006). When properly applied, sibship reconstruction and sibship‐based *N*_*b*_ estimation should prove to be useful tools for the conservation of vulnerable species.

## Data Archiving Statement

Biological and multilocus SNP data for individuals used in this study were stored on a Progeny (http://www.progenygenetics.com/) database server located at the Idaho Department of Fish and Game's Eagle Fish Genetics Laboratory (www.eaglefishgeneticslab.com). The final dataset used for this study, including both offspring and parent biological and SNP data, is also uploaded to and available at the standardized genetic repository www.FishGen.net.
