# Supplementary material for: Effective number of breeders from sibship reconstruction: empirical evaluations using hatchery steelhead
Source: Evol Appl. 2016 Oct 18;10(2):146–60. doi: 10.1111/eva.12433 (PMC5253425; doi:10.1111/eva.12433)
Supplement: Supplementary file 1 [file EVA-10-146-s001.pdf]

Supplementary material for:

Effective number of breeders from sibship reconstruction: empirical evaluations using hatchery steelhead

Michael W. Ackerman, Brian K. Hand, Ryan K. Waples, Gordon Luikart, Robin S. Waples, Craig A. Steele, Brittany A. Garner, Jesse McCane, Matthew R. Campbell

Title

Comparison of the two methods of Ne estimation from sibling assignments.

Introduction

Two methods for estimating Ne from sets of sibling assignments (PWOP: Waples 2011, and COLONY: Wang 2009) are compared. Here we discuss how they are conceptually related and show that the PWOP method is a special case of COPLONY method. The COLONY method can explicitly incorporate deviations from Hardy-Weinberg equilibrium and differences in sex ratio, while the PWOP method relies only on sibling relationships. This difference may not large in practice, as often these values are unknown and assigned default values by researchers, but see Wang (2009) and Wang (2016) for useful discussion.

In Supplemental Figures 1 and 2, using empirical data from the current study, we show that both the above methods give essentially the same estimates when applied to the same set of sibling assignment inferred from genetic data. Sibling assignments were estimated with COLONY2 (Jones and Wang 2010), assuming either a monogamous or polygamous mating system.

Definitions

N_e = Inbreeding effective population size, a measure of how the average inbreeding coefficient changes from one generation to the next.

N = Census size of the population during the parental generation.

N_p = Number of parents contributing at least one gamete to the next generation.

k_i = The number of offspring produced by the ith parent. This vector can include zeroes when indexed by $1 \rightarrow N$, or exclude zeroes when indexed by $1 \rightarrow N_p$.

$\overline{k}$ = The mean of the vector of k_i values.

$Var(\overrightarrow{k})$ = The variance of the vector of k_i values.

S = Number of (observed) offspring. Each offspring has two parents, so $S = \sum k_i/2$.

p_{same} = Chance that two random gametes in the offspring generation come from the same parent. In an ideal population, $p_{same} = \frac{1}{N}$. The chance that two random gametes are identical by descent in the previous generation is $P_{same}/2$ if the contribution of alleles by a parent is random and independent.

Equations

Crow and Denniston (1988):

$$N_e = \frac{\bar{k}N - 2}{\bar{k} - 1 + Var(\overrightarrow{k})/\bar{k}} \quad (1)$$

In equation (1) k_i contains **all** potential parents, even those with zero offspring. This equation assumes an equal sex ratio and progeny distribution.

Waples and Waples (2011) noted that (1) holds even when excluding individuals from the parental population that do not contribute offspring. Waples and Waples (2011) equation 2a:

$$N_e = \frac{\sum k_i - 1}{\frac{\sum k_i^2}{\sum k_i} - 1}. \quad (2)$$

They also note that this implies a sample of offspring can be used to estimate N_e by estimation of k_i (and crucially also $\sum k_i^2$).

As $\sum k_i = 2S$, the above equation can also be written as (Waples 2011, equation 2b):

$$N_e = \frac{2S - 1}{\frac{\sum k_i^2}{2S} - 1}. \quad (3)$$

Starting from the same vector of k_i values, we can equivalently calculate the chance that two gametes sampled at random without replacement share the same parent.

$$p_{same} = \frac{1}{2S(2S-1)} \sum_{i=1}^{N} (k_i(k_i - 1)), \quad (4)$$

where $2S(2S-1)$ is the number of gamete pairs and $\sum k_i(k_i - 1)$ is the number of gamete pairs sharing a parent, equal to $-2S + \sum k_i^2$. This allows equation 4 to be rewritten as:

$$p_{same} = \frac{\frac{\sum k_i^2}{2S} - 1}{2S - 1}, \quad (5)$$

which is simply the reciprocal of 3, providing the simple relationship:

$$N_e = \frac{1}{p_{same}}. \quad (6)$$

This connects the equations of Crow and Denniston (1988) and Waples (2011) to the chance that a random pair of gametes share a parent.

Wang (2009) addresses a very similar situation: "Equations for the effective size (N_e) of a population were derived in terms of the frequencies of a pair of offspring taken at random from the population being sibs sharing the same one or two parents".

Wang (2009) equation 10:

$$\frac{1}{N_e} = \frac{1+3\alpha}{4}(Q_1 + Q_2 + 2Q_3) - \frac{\alpha}{2}(\frac{1}{N_1} + \frac{1}{N_2}) \quad (7)$$

This equation directly addresses two potential departures from an ideal population; sex ratio and Hardy-Weinberg proportions. α is a measure of departure from Hardy-Weinberg equilibrium (i.e. F_{IS}), Q_1, Q_2, and Q_3 are the probabilities of a pair of offspring being paternal half-siblings, maternal half-siblings, and full-siblings, respectively. N_1 and N_2 are the number of male and female parents.

If we assume Hardy-Weinberg equilibrium ($\alpha = 0$), (7) becomes:

$$\frac{1}{N_e} = \frac{1}{4}(Q_1 + Q_2 + 2Q_3). \quad (8)$$

Wang (2009) equation 8:

$$p_{same\_mother} + p_{same\_father} = Q_1 + Q_2 + 2Q_3 \quad (9)$$

if we assume that the sex ratio is equal, this becomes:

$$\frac{2}{N} + \frac{2}{N} = \frac{4}{N} = Q_1 + Q_2 + 2Q_3 \quad (10)$$

and therefore:

$$\frac{Q_1 + Q_2 + 2Q_3}{4} = p_{same}. \quad (11)$$

Substitution into (8) leads to:

$$\frac{1}{N_e} = p_{same} \quad (12)$$

Which is the same as 6 and 2.

For a detailed discussion of the implications of these simplifying assumptions, see Wang (2009) and Wang (2016).

For an empirical example of the similarity of these methods, see the notebook at: `http://rwaples.github.io/PWOP-vs-SA/`

Figures

Figure 1: Scatterplot of N_e estimates with the PWOP and COLONY2 methods for sibling assignments assuming a monogamous mating structure. The Pearson correlation coefficient and p-value (as computed by scipy.stats.pearsonr) are reported.

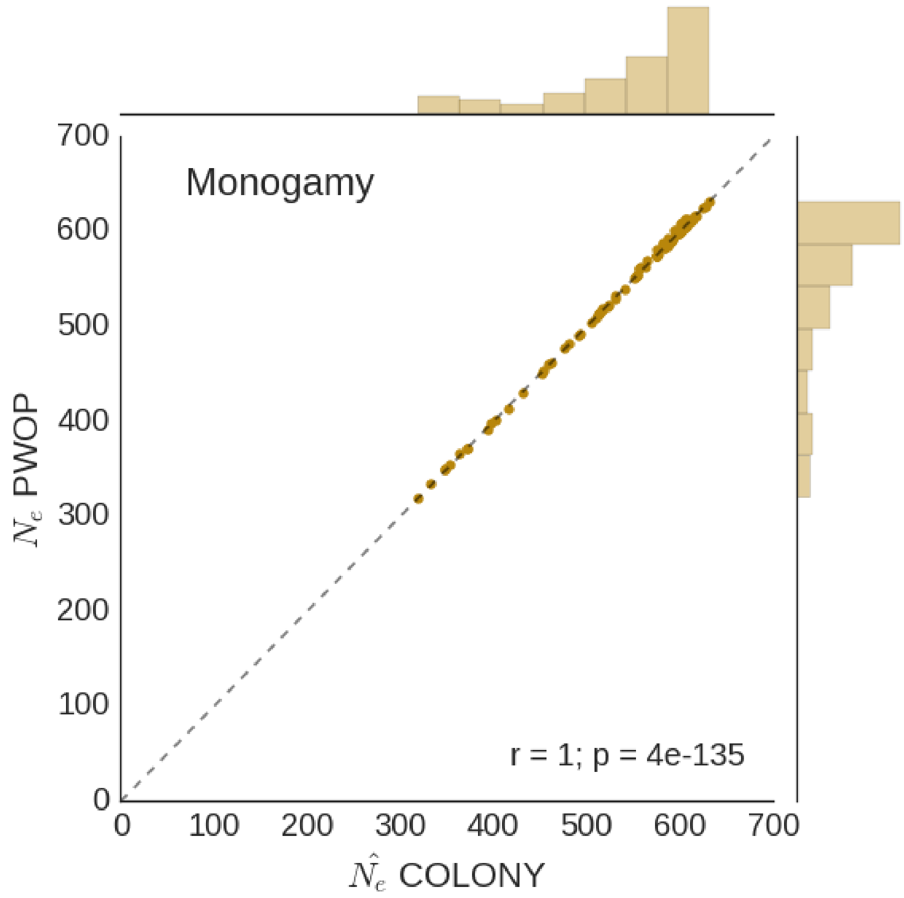

Figure 2: Scatterplot of N_e estimates with the PWOP and COLONY2 methods for sibling assignments assuming a polygamous mating structure. The Pearson correlation coefficient and p-value (as computed by scipy.stats.pearsonr) are reported.

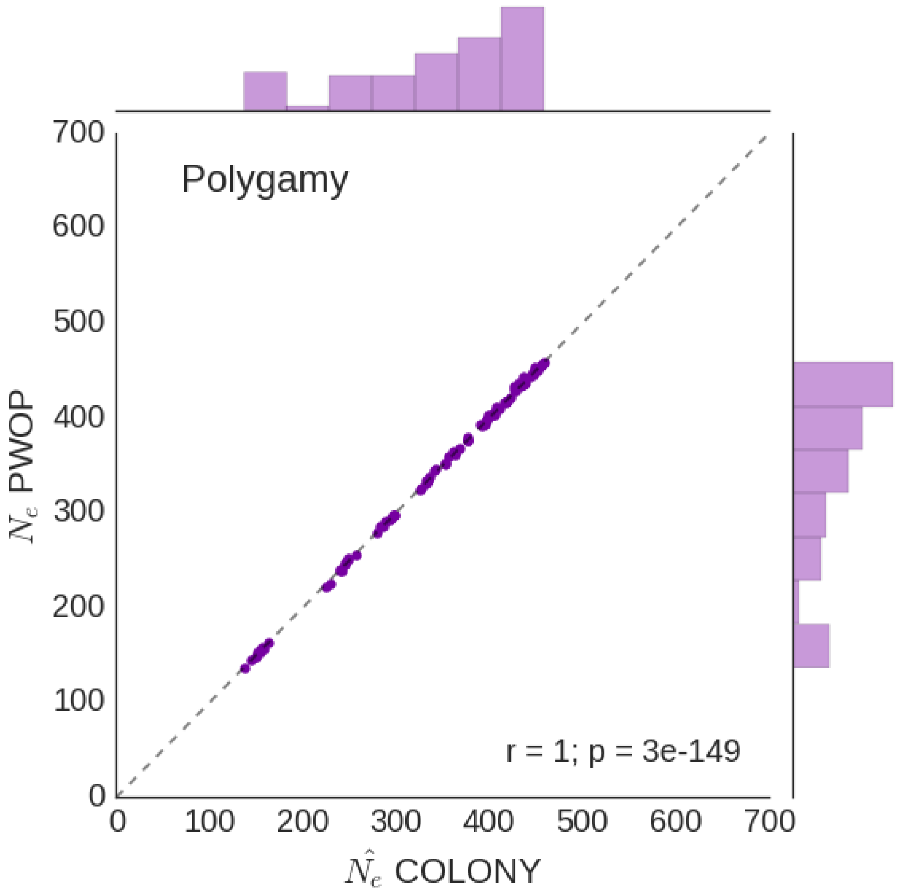

References

Crow, James F., and Carter Denniston. 1988. Inbreeding and variance effective population numbers. Evolution:482-495.

Jones, Owen R., and Jinliang Wang. 2010. COLONY: a program for parentage and sibship inference from multilocus genotype data. Molecular Ecology Resources 10.3:551-555.

Wang, Jinliang. 2009. A new method for estimating effective population sizes from a single sample of multilocus genotypes. Molecular Ecology 18.10: 2148-2164.

Wang, Jinliang. 2016. A comparison of single-sample estimators of effective population sizes from genetic marker data. Molecular Ecology. doi: 10.1111/mec.13725

Waples, Robin S., and Ryan K. Waples. 2011. Inbreeding effective population size and parentage analysis without parents. Molecular Ecology Resources 11: 162-171.
